# Combination of chemotherapeutic agents and biological response modifiers (immunotherapy) in triple-negative/Her2( +) breast cancer, multiple myeloma, and non-small-cell lung cancer

**DOI:** 10.1186/s43046-022-00159-8

**Published:** 2023-01-02

**Authors:** William Morse, Haroon Nawaz, Ayesha A. Choudhry

**Affiliations:** 1 Ross University School of Medicine, Miramar, USA; 2 Westside Regional Medical Center, Miramar, USA; 3 Fatima Jinnah Medical University, Lahore, Pakistan

**Keywords:** Immunotherapy, Chemotherapy, Cancer, Triple-negative breast, Myeloma, Non-small-cell lung cancer

## Abstract

**Hypothesis:**

Biological response modifiers (immunotherapy) in combination to chemotherapy are superior to that of chemotherapy in treatment of breast cancer (triple-negative/HER-2 ( +)), multiple myeloma, and non-small-cell lung cancer.

**Methods:**

This review article consists of a total of eighteen independent randomized controlled clinical trials ranging from phases one to three. Patients were randomly selected for immunomodulatory treatment or chemotherapy and assessed for a specific mutation expression that the immunomodulatory agent targets. Kaplan–Meier plots, swimmer plots, and bar graphs depict overall/progression-free survival, objective response, and clinical response rates. The data collected was assessed by using 95% confidence interval and a *p* value of 0.05. Patients were treated until disease progression.

**Results:**

Biological response modifiers (immunotherapy) resulted in significantly longer median progression-free survival in PD-L1-positive breast cancer (7.5 months compared to 5.0 months in control group), multiple myeloma (60.7% compared to 26.9% in the daratumumab and placebo groups, respectively), and in non-small-cell lung cancer (median progression-free survival was 10.3 months in the pembrolizumab group compared to 6.0 months in the chemotherapy group): higher complete responses in multiple myeloma (79% and 66% in the elotuzumab and control groups, respectively) and lower disease progression in PD-L1-positive non-small-cell lung cancer (62.1% of pembrolizumab versus 50.3% of chemotherapy patients had no disease progression at 6 months).

**Conclusion:**

Combination biological response modifiers (immunotherapy) and chemotherapy displayed benefit in overall/progression-free survival, response rate, duration of response, clinical benefit, and invasive disease-free survival in triple-negative/HER2-2( +) breast cancer, multiple myeloma, and non-small-cell lung cancer.

**Supplementary information:**

The online version contains supplementary material available at 10.1186/s43046-022-00159-8.

## Introduction

The Human Genome Project paved the means for current/future implications with regard to cancer treatment. Scientists had the ability to map out the whole human genetic code and discovered that cells are composed of a huge number of genes. Chemotherapeutic elements have been thoroughly utilized since the 1940s. They focus on rapidly dividing cells at certain phases in cellular replication, but do not differentiate between cancerous and healthy cells. This led to a spectrum of negative consequences resulting in nausea, myelosuppression, and hair loss. Chemotherapeutic regimens have limits, because of the hereditary variability within cancer cells, which allow them to have resistance to treatment. In 1986, scientists proposed a far more efficacious program which may be utilized exclusively or perhaps in conjunction with chemotherapy to fight cancer cells, referred to as biological response modifier (will be referred to as immunotherapy in this paper). This uses monoclonal antibodies as well as immune checkpoint inhibitors to target cancer cells. Immunotherapy targets unique cellular signaling conveyed in cancer cells. The combination of immunotherapy to that of chemotherapy allows cancer cells to be selectively targeted in multiple cellular replicative stages, resulting in longer median progression-free/overall survival with regard to triple-negative/HER 2( +) breast cancer, multiple myeloma, and non-small-cell lung cancer.

Triple-negative breast cancer is actually a phrase used to explain a cancer which does not express progesterone/estrogen receptors and under express a protein known as human epidermal growth factor receptor 2 (HER 2). This particular breast cancer is responsible for 10 to 15% of all the breast cancers and sadly tends to have a worse prognosis. This is because of the reality that it expands and spreads faster compared to some other cancers and is a lot more apt to come back again after treatment with regular chemotherapy. Based on the National Cancer Institute, the 5-year survival rate for localized, regional, or distant staging results is 91%, 65%, and 11%, respectively [1]. Chemotherapy continues to be the very first-line systemic treatment method, with international guidelines in support of utilizing single-agent anthracyclines or taxanes. Triple-negative breast cancer is much more typical in younger African American females than in persons of ethnic groups and other races, and it is frequently connected with visceral metastases. The median overall survival is roughly eighteen months or even less. Overall survival with this particular type of breast cancer has not changed in more than 20 years, demanding a more effective treatment protocol other than exercising traditional chemotherapeutic agents. VonMinckwitz et al. [2] (refer to evidence table in Supplementary information) proposed in a phase three study a more selective and effective therapy that targets vascular epidermal growth factor-A (VEGF-A) in combination to chemotherapy as opposed to using solely chemotherapy. In the triple-negative subtype group, the aim of adding Bevacizumab was to observe if there was any increased clinical pathological benefit. In the study, standard chemotherapy treatments epirubicin, cyclophosphamide, and docetaxel were compared with epirubicin, cyclophosphamide, docetaxel, and bevacizumab (a VEGF-A monoclonal antibody) in 663 metastatic triple-negative breast cancer patients, resulting in significant pathological complete responses. O’Shaughnessy et al. [3] (refer to evidence table in Supplementary information) observed in a phase two study that, in pathological and clinical features, triple-negative breast cancer was similar to BRCA 1 breast cancer. In triple-negative breast cancer, BRCA 1 (homologous-recombination DNA repair protein) was found to undergo somatic mutation, resulting in defects in DNA repair. The aim of the study was to target poly-adenosine diphosphate-ribose polymerase 1 (PARP1) with a PARP 1 inhibitor (iniparib) to prevent base excision repair in cancer cells. The combination of gemcitabine and carboplatin with iniparib (PARP 1 inhibitor) exhibited significant cytotoxic and antiproliferative effects as compared to gemcitabine and carboplatin in the 123-patient subgroup. Park et al. [4] (refer to evidence table in Supplementary information) discovered in a phase three trial that a tyrosine kinase inhibitor (neratinib) can be used to selectively inhibit ErbB and human epidermal growth factor receptor (HER 2 & HER 4), potentiating cytotoxic effects when given in combination to chemotherapy. The study also displayed activity of neratinib in patient subgroups that did not over express HER 2 & 4.

Multiple myeloma is a cancer of one’s plasma cells (antibody generating cells). Systemic therapy is comprised of chemotherapy (mephalan, cyclophosphamide, vincristine, etc.) as well as bone marrow transplant. Many individuals are treated with a blend of proteasome inhibitors, immunomodulatory elements, and monoclonal antibodies to extend drug opposition. The 5-year survival for multiple myeloma from 2009 to 2015 for localized, distant, along with combined stages are 74%, 51%, and 52%, respectively [5]. Due to the restricted treatment options for patients who have relapsed or refractory multiple myeloma to conventional chemotherapy and proteasome inhibition. A more innovative mode of treatment is essential for this incurable disease. In their preclinical data, Raje et al. [6] (refer to evidence table in Supplementary information) found that malignant cells express B cell malignant antigen (BCMA), which is a protein in the family of tumor necrosis factors. A chimeric antigen receptor T cell therapy (CAR-T) was developed using autologous T cells (bb2121) to target BCMA, resulting in complete tumor elimination and 100% survival in mouse models after receiving a single dose. A trial to treat patients who have been refractory to chemotherapy and proteasome inhibitors was performed in 2018. The study by Dimopoulos et al. [7] (refer to evidence table in Supplementary information) focused on comparing the combination of elotuzumab (monoclonal antibody targeting SLAMF7) with pomalidomide and dexamethasone in patients with relapsed and refractory multiple myeloma to that of pomalidomide and dexamethasone. By merging the immunotherapy agent with the chemotherapy regimen, specifically pomalidomide, they found a synergistic impact, leading to lower death rates. There is a subset of newly diagnosed multiple myeloma patients that were primarily treated with bortezomib (proteasome inhibitor), melphalan, and prednisone and are ineligible for autologous stem cell transplantation. Mateos et al. [8] (refer to evidence table in Supplementary information) contrasted the therapy described above with that of bortezomib, melphalan, prednisone, and daratumumab (human IgG kappa monoclonal antibody targeting CD38). This resulted in increased activity of cytotoxic T cells and a decrease in disease progression and death by 60%.

Lung cancer (small cell as well as non-small-cell) is the second most typical cancer in both females and males. Around 13% of lung cancers are small cell, and 84% are non-small-cell. The 5-year survival rate of non-small-cell lung cancer from 2009 to 2015 for localized, regional, and distant staging was 61%, 35%, and 6%, respectively [9]. Therapy depends on the driver mutation instead of the histologic subtype that may result in treatment resistance as well as a small array of therapeutic choices (cytotoxic chemotherapy). The addition of immunotherapy to chemotherapy regimens can prove to be a first line standard of care once more literature matures. Docetaxel is used as a second-line therapy to treat non-squamous non-small cell lung cancer that targets DNA polymerization in a non-selective manner, resulting in cessation of cell cycle replication. Non-small-cell lung cancer tumor cells express programmed death ligand-1 (PD-L1) and programmed death ligand-2 (PD-L2) which binds to programmed death receptor (PD-1) on activated T cells. This leads to weakening of T cell activation and allows the tumor cells to evade an immune response. Borghaei et al. [10] (refer to evidence table in Supplementary information) compared using nivolumab (IgG4 PD-1 immune checkpoint inhibitor antibody) to docetaxel in non-squamous non-small-cell lung cancer. It has been shown that non-small-cell lung cancer cells can express PD-L1with or without concomitant driver mutations. The patient subgroup with greater than 50% PD-L1 expression and no treatable driver mutation was primarily treated with platinum-based chemotherapy and pembrolizumab (PD-L1 antibody), resulting in longer overall survival with the targeted therapy. Hellmann et al. [11] (refer to evidence table in Supplementary information) conducted a trial utilizing nivolumab (IgG4 PD-1 immune checkpoint inhibitor antibody) with ipilimumab, which is an anti-cytotoxic lymphocyte antigen 4 antibody (CTLA-4 antibody). This combination works by selectively inhibiting a pathway that would normally downplay an individual’s immune system, resulting in cancer cell evasion from an immune response. The study found a significant increase in progression-free survival in the patient subgroup treated with nivolumab and pembrolizumab that may lead one to think about the significance of a future trial of adding a chemotherapeutic agent to this effective treatment. Throughout this paper one will be enlightened that immunotherapy in conjunction with chemotherapy is better to that of chemotherapy in treatment of breast cancer (triple-negative/HER 2 ( +)), multiple myeloma, and non-small-cell lung cancer.

## Methods

The articles were gathered via New England Journal of Medicine and American Cancer Society search engines. The main database used to gather findings in this literature review was from New England Journal of Medicine. One method of search strategy for obtaining breast cancer literature involved using keywords: (Breast) AND (cancer) AND (triple-negative) AND (HER 2-positive) (51 articles found). Another strategy involved using “triple-negative/ HER 2-positive breast cancer” (28 articles found). The filters used in all searches encompassed: past 10 years (2010–2020), specialty (hematology/oncology), research articles, and most relevant. The study population of interest were female patients with triple-negative with/without HER 2( +) breast cancer who had been treated with combination immunotherapy and chemotherapy. Other inclusion criteria in all searches dealt with obtaining original immunotherapy articles and previous/on-going clinical trials. Criteria used in all searches to exclude articles from this paper included review articles, perspective articles, commentary articles, non-immunotherapy trials, and clinical cases. Keywords applied in New England Journal of Medicine search engine for multiple myeloma literature involved: (multiple myeloma) and (cancer) AND (immunotherapy) and (chemotherapy) (18 articles found). Another search strategy used was “multiple myeloma” (1424 articles found). The same filters were applied as previously mentioned (35 articles found). The study population of interest were individuals that had untreated or relapsed and refractory multiple myeloma to standard treatment. The inclusion and exclusion criteria were the same as previously mentioned. Keywords applied in New England Journal of Medicine search engine for non-small-cell lung cancer literature involved: (non-small-cell) and (cancer) AND (immunotherapy) (49 articles found). Another search strategy used was “non-small-cell lung cancer” (317 articles found). The same filters were applied as previously mentioned (52 articles found). The study population consisted of non-small-cell lung cancer patients that expressed a driver mutation and treated with combination immunotherapy and chemotherapy. One can refer to the Supplementary information and locate “Evidence table,” which outlines the key findings of the eighteen articles that met these criteria.

## Results

The application of nab-paclitaxel and atezolizumab in the advanced triple-negative breast cancer study (refer to clinical trials Table 1 in the “Results” section) was comprised of utilizing a humanized monoclonal antibody (atezolizumab) that binds to programmed death ligand 1 (PD L1), resulting in enhanced overall survival and progression-free survival. Selection criteria could be seen in Additional file 1: Figure S1A. A total of 902 individuals (intention-to-treat) had been enrolled in more than 246 sites in forty-one countries. There seemed to be a total of 451 people randomly assigned to each intention to treat group in Additional file 1: Table S1A. The PD L1 subgroup was comprised of a total of 369 individuals, which was symbolic of the intention-to-treat population [12]. The median length of treatment in the atezolizumab (treatment dose of 1980 ± 1303.1 mg/m^2^) and nab-paclitaxel groups were 24.1 as well as 22.1 weeks, respectively. The median length of treatment in the placebo-nab-paclitaxel group (treatment dose of 1764.4 ± 1238.3 mg/m^2^) was 22.1 weeks and 21.8 weeks in nab-paclitaxel group [12] (refer to evidence table in the Supplementary materials).

**Table 1:** An overview of the clinical trials used throughout the results and discussion will be found below

Clinical trial	Target of therapy	Mechanism of therapy	Phase of trial	Endpoints
**Triple-negative breast cancer**				
Atezolizumab and nab-paclitaxel in advanced triple-negative breast cancer	Programmed death ligan-1 (PD-L1)	Selectively targets PD-L1	Phase 3	Progression-free survival & overall survival
Sacituzumab govitecan-hziy in refractory metastatic triple-negative breast cancer	Human trophoblast cell-surface anti- gen 2 (Trop-2)	SN38 (active metabolite of irinotecan) coupled to a humanized antitrophoblast cell-surface antigen 2 (Trop-2) monoclonal antibody hRS7 IgG1κ through the cleavable CL2A linker	Phase 1/2	Safety, objective response rate, duration of response, clinical benefit rate, progression-free survival, overall survival
Complete response of chemo-refractory metastatic metaplastic breast cancer to paclitaxel-immunotherapy combination	Programmed death ligan-1 (PD-L1)	Anti-PD-L1 antibody (durvalumab)	Phase 1/2	Safety, duration of response, objective response rate
**HER-2-positive breast cancer**				
Adjuvant pertuzumab and trastuzumab in early HER2-positive breast cancer	Human epidermal growth factor receptor-2	Humanized monoclonal anti-body that binds to the dimerization domain, inhibiting HER2 heterodimerization with other HER family receptors	Phase 3	Invasive-disease-free survival, recurrence of ipsilateral invasive breast tumor, recurrence of ipsilateral locoregional invasive disease, overall survival, disease-free survival
**Multiple myeloma**				
Elotuzumab therapy for relapsed or refractory multiple myeloma	Signaling lymphocytic activation molecule F7 (SLAMF7)	Activating natural killer cells and mediate antibody-dependent cell-mediated cytotoxicity through the CD16 pathway	Phase 3	Progression-free survival and the overall response rate
Oral selinexor–dexamethasone for tripleclass refractory multiple myeloma	Exportin-1 (XPO1)	Selective inhibitor of nuclear export that binds to Cys528 in the cargo-binding pocket of XPO1	Phase 2b	Overall response
Daratumumab, bortezomib, and dexamethasone for multiple myeloma	CD38	Human IgGκ monoclonal antibody that targets CD38	Phase 3	Progression-free survival
**Non-small-cell lung cancer**				
Rociletinib in EGFR-mutated non–small-cell lung cancer	T790M mutation	Mutant-selective covalent inhibitor of EGFR with T790M mutation	Phase 1–2	safety, side-effect profile, and pharma- cokinetic characteristics, progression-free survival, objective response rate
Pembrolizumab versus chemotherapy for PD-L1-positive non-small-cell lung cancer	Programmed death ligand-1 (PD-1)	Humanized monoclonal antibody against programmed death 1 (PD-1)	Phase 3	Progression-free survival, overall survival, objective response rate, safety
Durvalumab after chemoradiotherapy in stage III non–small-cell lung cancer	Programmed death ligand-1	Human IgG1 monoclonal antibody	Phase 3	Progression-free survival, overall survival, objective response rate, duration of response, safety

Additional file 1: Table S2A signifies secondary efficacy results. As seen in Additional file 1: Figure S2A, the overall median survival of the PD-L1 subgroup was 25 months (atezolizumab-nab-paclitaxel group) and 15.5 months (placebo-nab-paclitaxel group) represented in the Kaplan–Meier plots. In the intention-to-treat group, the speed of objective response was 56% in the atezolizumab population when compared to 45.9% in the placebo group. About 7.1% of individuals in the atezolizumab group had a complete response to 1.1% of people taking placebo-nab-paclitaxel. The PD L1( +) subgroup response rate as well as complete response was 58.9% and 10.3% in the atezolizumab treated group compared to 42.6% and 1.1% in the placebo population. The median response duration for the population intended for treatment was 7.4 months (atezolizumab-nab-paclitaxel group) and 5.6 months (placebo-nab-paclitaxel group). The median period of response was 8.5 months in the PD-L1 group (atezolizumab-nab-paclitaxel group) and 5.5 months in the PD-L1 group (placebo-nab-paclitaxel group). Grade three or four adverse effects occurred more often in the atezolizumab-nab-paclitaxel group (48.7%) than in the placebo-nab-paclitaxel group (42.4%), with neutropenia, anemia, and diarrhea occurring more often in the two groups [12].

The application of sacituzumab govetican-hziy in refractory metastatic triple-negative breast cancer was comprised of utilizing a humanized anti-trophoblast cell surface antigen two (Trop 2) monoclonal antibody hRS7 IgG1κ via the cleavable CL2A linker opposing human trophoblast cell surface antigen two (Trop 2), resulting in improved progression-free/overall survival (refer to clinical trials Table 1 in the “Results” section). The median period of exposure was 5.1 months among the 108 individuals (average of 9.6 cycles). Neutropenia, anemia, and diarrhea were seen as adverse effects of grade three or four. Additional file 1: Figure S1B reveals the responses of 108 people with metastatic triple-negative breast cancer being treated with sacituzumab govetican-hziy [13]. The response and complete response rates are 33.3% as well as 2.8%, respectively. Clinical benefit (including stable illness of 6 months) was 45.4%. Part B of Additional file 1: Figure S1B displays a swimmer plot comprising of durability and onset of the thirty-six individuals that had an objective response. The median time to response as well as duration of response was 2.0 months and 7.7 months (95% confidence interval [CI], 4.9 to 10.8). The response rate and median response period were 34.3% ([95 percent CI, 25.4 to 44.0]) and 9.1 months ([95% CI, 4.6 to 11.3]), respectively. Overall, age, onset of disease, and prior therapy did not play a major role in response rates in patients. Part C of Additional file 1: Figure S1B shows progression-free survival at 6 and 12 months were 41.9% and 15.1%. The median overall survival was 13 months (95% CI, 11.2 to 13.7). At 6 and 12 months, the probability of survival was 78.5% and 58.3%, respectively. Additional file 1: Figure S2B illustrates the individuals being treated with sacituzumab govetican-hziy compared to individuals that had earlier anticancer therapy had a median length of treatment of 5.1 months compared to 2.5 months, that sheds light upon the clinical task, and absence of cross resistance with anti-body drug conjugate [13] (refer to evidence table in Supplementary information).

Because of the absence of effective standard treatment to control metastatic metaplastic triple-negative breast cancer a patient was provided enrollment in a clinical chemo-immune trial. The Durvalumab conjunction with paclitaxel in therapy of chemo-refractory metastatic metaplastic breast cancer addressed utilizing a PD L1 antibody in PD L1( +) individuals (refer to clinical trials Table 1 in the “Results” section). This particular study was comprised of a 49-year-old premenopausal female who received four cycles of capecitabine and confirmed disease that is progressive with an increase in number and size of pulmonary/mediastinal lymph nodes in Additional file 1: Figure S3B component A. Additional file 1: Figure S3B established pelvic metastasis in the right iliac bone as well as femoral head [14]. The CT scan demonstrates total clearance of pulmonary/chest wall structure lesions and resolution of the associated soft tissue part of the lytic bone in Additional file 1: Figure S2B component B. The bone scan indicates normalization of uptake within the right iliac bone in Additional file 1: Figure S3B (Al sayed., et al. 2019) (refer to evidence table in Supplementary information).

HER 2( +) breast cancer managed with adjuvant pertuzumab (binds to domain 2 of HER 2 receptor) and trastuzumab (binds to domain 4 of the HER 2 receptor) in HER 2-positive breast cancer (refer to clinical trials Table 1 in the “Results” section) has proven benefit regarding invasive-disease-free survival, that can be found in Additional file 1: Figure S1C. This study was comprised of a total of 4805 individuals randomly selected, with 2400 individuals in the pertuzumab as well as 2405 individuals in the placebo. Disease incidents were lower in the pertuzumab group (7.1%) than in the placebo group (8.7%), as can be shown in Additional file 1: Figure S1C. A 3-year invasive-disease-free survival was 94.1% in the pertuzumab group when compared to 93.2% in placebo group (95% confidence interval [CI], 0.66 to 1.00; *P* = 0.045). Locoregional and distant recurrences in the pertuzumab as well as placebo groups had been 4.7% as well as 1.1% and 5.8% as well as 1.4%, respectively. The amount of invasive disease incidents was 3.6% in node-negative individuals in the pertuzumab group when compared to 3.2% in placebo. Node-positive demonstrated 9.2% in the pertuzumab group as well as 12.1% in placebo group had invasive disease incidents. Grade three or four diarrhea, anemia, or neutropenia were the most common adverse events in both groups, but diarrhea was more severe in the pertuzumab group (9.8%) than in the placebo group (3.7%) (Von Minckwitz et al. 2018) (refer to evidence table in Supplementary information).

From 2011 to 2012, 646 individuals had been randomly selected in 168 diverse sites worldwide underwent remedy with elotuzumab (monoclonal antibody which binds to SLAMF7) for relapsed or refractory multiple myeloma (refer to clinical trials Table 1 in the “Results” section). The rate of progression-free survival was 68% (95% confidence interval [CI], 63 to 73) and 41% (95% CI, 35 to 47) in the elotuzumab group as opposed to 57% (95% CI, 51 to 62) and 27% (95% CI, 22 to 33) in the control group at 1 and 2 years. Median progression-free survival was 19.4 months (95% CI, 16.6 to 22.2) in the elotuzumab individuals as compared to 14.9 months (95% CI, 12.1 to 17.2) in the control group, resulting in a 30% reduction in disease progression or death that can be viewed in Additional file 1: Figure S1D [15]. The elotuzumab group was shown to have a 32% relative risk reduction in progression-free survival and to have substantially increased progression-free survival in patients who were diagnosed for 3.5 years or longer. Compared to the control group, the independent review committee considered that there were less complete responses in the elotuzumab group. In contrast to 28% of the control, a partial or better response was seen in 33% of the elotuzumab group. The median survival was 26 months in the elotuzumab group as opposed to 17.3 months in the control group. Overall response rates were 79% (95% CI, 74 to 83) and 66% (95% CI, 60 to 71) in the elotuzumab as well as control group, respectively. Responses had been discovered to be longer in the elotuzumab group (21 months; 95% CI, 18 to 27) compared to control (17 months; 95% CI, 15 to 19). The median treatment period was 17 months (elotuzumab group) and 12 months (control group), respectively. Neutropenia and lymphocytopenia of grades three or four were 34% and 77% in the elotuzumab group and 44% and 49% in the control group [15] (refer to evidence table in Supplementary information).

The oral use of selinexor and dexamethasone for triple class refractory multiple myeloma (refer to clinical trials Table 1 in “Results” section) consisted of 122 individuals with progressive myeloma at time of enrollment. They had been treated with selinexor that selectively targets exoportin 1, resulting in a more efficacious response rate and overall/progression-free survival. A partial response or better was found in 26% (95% confidence interval [CI], 19 to 35) of individuals, a minimum response or better was seen in 39% (95% CI, 31 to 49) of individuals, and 2% had a complete response. It took patients 4.1 weeks to obtain a median response. The median progression-free survival was 3.7 months (95% CI, 3.0 to 5.3), and median overall survival was 8.6 months (95% CI, 6.2 to 11.3) as seen in Additional file 1: Figure S1E. In individuals that had a minimal/partial response or better had a median overall survival of 15.6 months as seen in Additional file 1: Figure S1E. In 59%, 44%, and 22% of those treated with oral-selinexor, grade three or four thrombocytopenia, anemia, and neutropenia were observed, respectively. In the patient population with baseline thrombocytopenia, however, thrombocytopenia was more common [16] (refer to evidence table in Supplementary information).

A total of 498 individuals had been enrolled in the daratumumab, bortezomib, and dexamethasone for multiple myeloma study (refer to clinical trials Table 1 in “Results” section). 251 individuals were randomly assigned to the daratumumab group and 247 to the control group. The median lines of treatment that individuals had just before the analysis was two. Daratumumab is a human IgGκ monoclonal antibody which targets CD38, resulting in improved progression-free survival. Progression-free survival was 60.7% (95% confidence interval [CI], 51.2 to 69.0) as compared to 26.9% (95% CI, 17.1 to 37.5) in the daratumumab as well as placebo groups, seen in Additional file 1: Figure S1F. In the daratumumab group, median progression-free survival was not reached (95% CI, 12.3 to non-estimated) and was achieved in the control group (7.2 months) (95% CI, 6.2 to 7.9). There seemed to be a 61.4% decline in death or disease progression in the daratumumab group than in the control as seen in Additional file 1: Figure S1F. After 12 months, 65.4% (95% CI, 56.1 to 74.8) as opposed to 28.8% (95% CI, 17.8 to 39.8) were totally free from disease progression, displayed in Additional file 1: Figure S1F. Daratumumab displayed a substantially higher overall response rate, partial response rate, and complete response rate of 82.9% (*P* < 0.001), 59.2% (*P* < 0.001), and 19.2% (*P* = 0.001), compared to 63.2% (*P* < 0.001), 29.1% (*P* < 0.001), and 9% (*P* = 0.001) in the control group. In the daratumumab group, the median time and period of response were more favorable (0.9 months and 11.5 months) relative to the control group (1.6 months and 7.9 months). The 12-month progression-free survival was 77.5% (daratumumab group; 95% CI, 65.2 to 86.0) and 29.4% (daratumumab group; 95% CI, 65.2 to 86.0) for patients who previously received one line of therapy prior to enrollment in the study (control group; 95% CI, 12.5 to 48.7). Grade three or four adverse reactions were higher in the daratumumab group (76.1%) than in the control group (62.4%). These include thrombocytopenia (45.3% as compared to 32.9%), anemia (14.4% as compared to 16%), and neutropenia (12.8% compared to 4.2%) [17] (refer to evidence table in Supplementary information).

The application of rociletinib in the EGFR mutated non-small-cell lung cancer study (refer to clinical trials Table 1 in “Results” section) consisted of 130 people at ten global centers. The median number of previous treatments were four. The EGFR T790M mutation could be selectively targeted by rociletinib (mutant selective covalent inhibitor of EGFR with T790M mutation). Before patients were able to join the study, T790M expression had to be confirmed through tumor biopsy. Initially, patients in the rociletinib group received a starting dose of 150 mg once daily and subsequently received 900 mg twice daily, resulting in a continuous objective response in this study arm, as shown in Additional file 1: Figure S1G. Forty-six people with centrally confirmed T790M ( +) tumors had a response rate of 59% (95% confidence interval [CI], 45 to 73) and disease control rate of 93%. The progression-free survival was 13.1 months (95% CI, 5.4 to 13.1). Response/disease control rates were 29% (95% CI, 8 to 51) as well as 59% in individuals with T790M (-) core testing. The progression-free survival was 5.6 months (95% CI, 1.3 to not reached) in the T790M (-) group. The most common grade three event was hyperglycemia, which was controlled by patients being administered hypoglycemic agents, as well as, tapering down the dose of rociletinib (48% of patients had a dose reduction) (Sequist., et al. 2015) (refer to evidence table in Supplementary information).

The pembrolizumab versus chemotherapy for PD-L1-positive non-small-cell lung cancer study (refer to clinical trials Table 1 in “Results” section) consisted of randomly selecting 154 individuals in the pembrolizumab group as well as 151 individuals in the chemotherapy group. This particular cancer has a mutation in programmed death ligand 1 and may be addressed with pembrolizumab (humanized monoclonal antibody against programmed death one (PD 1)). The number of median treatment cycles in the pembrolizumab group was substantially greater than in the chemotherapy group (10.5 vs 4 cycles). After disease progression, about 43.7% of chemotherapy patients crossed into the pembrolizumab group and about 57.6% continued to receive pembrolizumab after the cut-off time. Median progression-free survival was 10.3 months (95% confidence interval [CI], 6.7 to not reached) in the pembrolizumab group compared to 6.0 months (95% CI, 4.2 to 6.2) in the chemotherapy group as seen in Additional file 1: Figure S1H. 62.1% (95% CI, 53.8 to 69.4) of pembrolizumab as opposed to 50.3% (95% CI, 41.9 to 58.2) of chemotherapy individuals had no disease development at 6 months. Progression-free survival in all subgroups in the pembrolizumab arm was substantially longer. Of the patients treated with pembrolizumab, about 44.8% (95% CI, 36.8 to 53.0) had an objective response rate, compared with 27.8% (95% CI, 20.8 to 35.7) in the chemotherapy group. While both groups had a median response time of 2 months, the median response period was not reached in the pembrolizumab group and was 6.3 months in the chemotherapy arm. The overall survival in the pembrolizumab group was considerably longer than in the chemotherapy group, as shown in Additional file 1: Figure S2H. Adverse events such as diarrhea (14.3%), fatigue (10.4%), and pyrexia (10.4%) in the pembrolizumab group and anemia (44%), nausea (43.3%), and fatigue (28.7%) in the chemo group were experienced in about 73.4% of pembrolizumab patients and 90% of chemo patients [18] (refer to evidence table in Supplementary information).

The inclusion of durvalumab after chemoradiotherapy in stage III non-small-cell lung cancer resulted in a major advantage for individuals regardless of PD L1 expression. Durvalumab is a human IgG1 monoclonal antibody which targets PD L1, resulting in T cells destroying and recognizing tumor cells. 473 individuals were randomly selected to the durvalumab group, as well as 236 individuals were in the placebo (chemotherapy) group. The durvalumab group had a median of twenty infusion cycles compared to only fourteen infusions in the placebo group. The progression-free survival rates of 12 and 18 months in the durvalumab group were 55.9% (95% CI, 51.0 to 60.4) and 44.2% (95% CI, 37.7 to 50.5) compared to 35.3% (95% CI, 29.0 to 41.7) and 27% (95% CI, 19.9 to 34.5) in the placebo group. Median progression-free survival was 16.8 months (95% confidence interval [CI], 13.0 to 18.1) in durvalumab group compared to 5.6 months (95% CI, 4.6 to 7.8) in placebo as seen in Additional file 1: Figure S1I. The median moment to death/distant metastasis was 23.2 months (95% CI, 23.2 to not reached) as compared to 14.6 months (95% CI, 10.6 to 18.6) with placebo. In the durvalumab group (28.4%; *P* < 0.001), the objective response rates were more significant than in the placebo (16%; *P* < 0.001). In comparison to placebo, the durvalumab arm had a significantly greater rate of adverse effects (96.8 vs 94.9%). The most common grade three or four adverse effects were pneumonia (4.4% in durvalumab group compared to 3.8% in placebo) [19] (refer to evidence table in Supplementary information).

## Discussion

Schmid et al. [12] discovered that tumor infiltrating immune cells express programmed death ligand one (PD L1), which prevent anticancer immune response. A drug known as atezolizumab targets PD L1 to prevent interaction with B7-1 and pd-1 receptors (co-stimulatory cell surface protein), preventing more T cell suppression. Administering nab-paclitaxel and atezolizumab in advanced triple-negative breast cancer as first line treatment led to a substantially longer progression-free survival than was observed in the placebo-nab-paclitaxel in the intention-to-treat and PD L1-positive tumor groups. The threshold for declaring a statistical edge for atezolizumab-nab-paclitaxel in the intention-to-treat group in overall survival was not passed, and proper testing was not performed in PD L1( +) subgroup, resulting in an increase in median overall survival in the intention-to-treat and PD L1( +) subgroups. A clinical benefit of PD L1( +) subgroups was found in median progression-free survival (7.5 months with atezolizumab-nab-paclitaxel vs. 5.0 months with placebo-nab-paclitaxel), overall survival (25.0 months atezolizumab vs. 15.5 months with placebo), and unbiased response rate (58.9% with atezolizumab vs. 42.6% with placebo) in the atezolizumab group. The safety profile was in line with observations from other atezolizumab chemotherapy mixture trials, resulting in no new negative event observations. Confounding variables remained significantly small because of balancing the clinical features at baseline and post protocol therapies in the trial organizations. The PD L1( +) patient advantage with obtaining atezolizumab-nab-paclitaxel in this trial provides proof of the efficacy attained in this specific affected person subgroup, concerning immunotherapy. This study shows the potential of introducing immunotherapy to chemotherapy in terms of response rates and survival, which can serve as a more favorable standard of care option for qualifying triple-negative patients with breast cancer, even though the adverse hematological events (neutropenia and anemia) along with diarrhea were slightly higher. One can observe that the benefits outweigh the risks when it comes to achieving a clinical response to treatment. For future implications, it is crucial to take into account individuals PD L1 expression in metastatic triple-negative breast cancer for therapy [12].

Bardia et al. [13] discovered that the Trop 2 transmembrane calcium signal transducer is over expressed in triple-negative breast cancer (85% of tumors). A stage 1/2 individual team, multicenter trial required 108 individuals that received sacituzumab govitecan-hziy after receiving a minimum of two earlier anti-cancer treatments for metastatic triple-negative breast cancer. This antibody drug conjugate fuses a humanized monoclonal antibody which targets Trop two (human trophoblast cell surface antigen two) with SN 38 (topoisomerase 1 inhibitor), that is conjugated to an antibody by a cleavable linker. This allows for intracellular uptake of SN-38 and killing of adjacent cells via extracellular release of its contaminated metabolite. Among individuals that received a median number of three prior therapies before administration of sacituzumab govitecan-hziy had a response rate, median period of reaction, progression-free survival, and overall survival of 33.3%, 5.5 months, 7.7 months, and 13 months, respectively, in metastatic triple-negative breast cancer. The duration of therapy with sacituzumab govitecn-hziy (5.1 months) was longer than with previous antitumor treatment (2.5 months) that further offers activity in individuals with hard-to-treat metastatic triple-negative breast cancer. The tiny patient population led to large confidence intervals, and clinical results observed in subgroups are actually poor, which results to information interpretation to be seen with extreme caution. The high expression of Trop 2 is linked with a bad prognosis in triple-negative breast cancer and that suggests usage of sacituzumab care is actually a logical approach in this particular population. The cytotoxic element of sacituzumab govitecan-hziy is SN 38 (irinotecan metabolite), that is a hundred to thousand times more cytotoxic than exclusively irinotecan (chemotherapy). The sacituzumab group had a much better side effect profile as opposed to the placebo group. Immediate comparison along with other chemotherapeutic agents could not be done because of the study is non-comparative design. The mechanism of action behind this treatment when compared to solely chemotherapy clearly provides a new standard of care in Trop 2-positive individuals, due to the immunotherapy acting as a selective key to bind to the trop 2 protein on cancer cells, that allows a channel to open for chemotherapy to enter and destroy the pathological cells. Similar adverse effects were more prominent in the sacituzumab group as seen in the Schmid et al. [12] study, which were neutropenia, anemia, and diarrhea. Future implications pertaining to Trop 2-targeted immunotherapy look very promising once more data is conducted regarding lock and key mechanism of action with combination chemotherapy. The main endpoint was the response rate, since it is much less subject to bias than to progression-free survival in a single-group trial which has been utilized for accelerated approval in some other oncology trials [13].

Metaplastic breast cancer is a rare subtype of breast cancer with under a 20% response rate to systemic chemotherapy. Al Sayed et al. [14] discovered expression of PD L1 on this particular cancer variant, which led them to carry out a clinical trial, that shown a total result in a patient with metaplastic breast cancer to paclitaxel durvalumab (anti-PD-L1 antibody) mixture. Metastatic metaplastic breast cancer is treated in the same fashion as triple-negative breast cancer but is actually regarded as to be chemo resistant with a poorer prognosis. This particular cancer has pathological functions of neoplastic epithelium changing into mesenchymal-like components, resulting in the upregulation of PD L1 expression which allows the evasion of cancer cells. This particular individual achieved total response in bone, chest wall, lymph nodes, and lung after ten cycles with durvalumab. The information must be seen with caution because this is the very first case of paclitaxel and combination durvalumab to treat metastatic metaplastic breast cancer. Since there is no current cure and extremely low response rate to treatment, this case is even more important, because of the complete pathological response that was seen. The Schmid et al. [12] study used atezolizumab (PD L1 antibody), which can perchance open a door for future implications of considering trials that test whether there is any benefit of using a preferred combination immunotherapy/chemotherapy protocol over another regarding specific PD L1 ( +) tumors.

Human epidermal growth factor receptor 2 is conveyed in HER 2( +) breast cancer and has long been utilized as a target for immunotherapy. The Von Minckwitz et al. [20] study dealt with including pertuzumab (binds to domain 2 of HER 2 receptor) to the present routine of adjuvant trastuzumab (binds to domain 4 of the HER 2 receptor) as well as chemotherapy in epidermal growth factor receptor two (HER 2) breast cancer to induce antibody-dependent cell-mediated cytotoxicity. Patients with HER 2( +) breast cancer sought enhancement with trastuzumab and combination pertuzumab with chemotherapy. The median time of follow-up was 45.4 months, which might have been too short for a complete assessment of the impact size. Various other analyses for this study have as much as 10 years of follow-up, and also in 2.5 years, there will be another analysis. This trial was a big, adequately powered, double blind, placebo-controlled phase 3 trial. During the recruitment stage of the study process, amendments have been positioned in order to restrict individuals with node-negative illness as well as to boost the sample size, in order to yield an individual population with nodal status distribution. The efficacy of various other remedies remains unknown, due to the 1-year trial this particular study had; on-going studies are finding if after 6 months of therapy with neoadjuvant pertuzumab will require treatment after surgery. A significant benefit in invasive-disease-free survival was seen in the pertuzumab group when compared to placebo as well as having a lower rate of disease recurrence (Von Minckwitz et al. 2018). This case can serve as an example for future implications of combining two or more immunotherapies with chemotherapy to achieve a potentially more efficacious response. One can question if similar approaches could be applied to the Schmid et al. [12], Bardia et al. [13], and Al Sayed et al. [14] studies with respect to their targeted modalities. Similar adverse events were present in this trial to that of the aforementioned ones, being neutropenia, anemia, and diarrhea.

The Lonial et al. [15] study discovered that more than 95% of bone marrow myeloma cells express signaling lymphocytic activation molecule F7 (SLAMF7) cell surface glycoprotein. In 2015, this stage three clinical trial was comprised of 321 individuals receiving mixture elotuzumab (an immunostimulatory monoclonal antibody targeting signaling lymphocytic activation molecule F7 (SLAMF7)) to lenalidomide as well as dexamethasone to treat individuals with relapsed or refractory multiple myeloma. Elotuzumab targets SLAMF7 glycoprotein that is conveyed on natural killer and myeloma cells. This results in initiating natural killer cells and mediating antibody-dependent cell-mediated cytotoxicity via the CD16 pathway. Combination elotuzumab, lenalidomide, and dexamethasone therapy for relapsed or refractory multiple myeloma led to an increase in progression-free and overall survival outcomes, compared to lenalidomide and dexamethasone treatment. SLAMF7 was discovered to be primarily conveyed on plasma cells (normal or malignant), natural killer cells, along with other immune cells, but was not conveyed on other typical tissue. Elotuzumab works via two mechanisms, one being through antibody-dependent cell-mediated cytotoxicity. The other pathway is via an independent method which does not involve Fc portion binding and immediate activation of CD16 (marker for antibody-dependent cell-mediated cytotoxicity)-deficient natural killer cells through SLAMF7 receptors. This led to a distant relative reduction of 30% in disease progression or death when compared with the control group. Follow-up for survival outcomes is on-going. The advantage to disease progression was established by several sensitivity analyses. There seemed to be a thirteen-percentage point difference in the general response rate, but fewer entire responses in the elotuzumab group when compared with the control group. Alterations in lymphocyte and natural killer cellular trafficking could be represented by the lymphocytopenia which was found in the elotuzumab group. There was also no proof of autoimmunity, which could be connected with immunostimulatory agents. The clinical efficacy confirms the two-action usage of elotuzumab treatment in multiple myeloma. Future implications regarding combination SLAMF7-targeted therapy with chemotherapy look promising as it seems to be similar to the approaches taken in triple-negative breast cancer, by preventing downregulation of one’s immune response as well as potentiating the effects of the cytotoxic chemotherapy.

Chari et al. [16] discovered a nuclear exporter of tumor suppressor proteins (exportin 1) found to be overexpressed in multiple myeloma cells. A report involved treating 122 individuals with myeloma that are refractory to present therapeutics with Selinexor (exportin 1 inhibitor). This resulted in nuclear accumulation as well as activation of tumor suppressor proteins, inhibition of nuclear component kappa beta, and decrease in oncoprotein messenger RNA (mRNA) interpretation, leading to apoptosis of malignant hematologic as well as sound tumor cells. The application of oral selinexor-dexamethasone for triple-class refractory multiple myeloma had 26% of individuals with a partial effect or even better. All of these individuals had disease that is progressive, with 21% having disease progression or maybe the disease of theirs could not be evaluated for a response. The efficacy was constant among almost all subgroups that had a result and in individuals with high-risk cytogenetic abnormalities (53% of individuals). The individual population selected had a median of seven prior therapeutic regimens, including a median of ten antimyeloma agents, that would be in line with the increasing population of individuals that have run out therapeutic choices and really desire treatment. The preclinical information of selinexor shown enhancement of IκB and that supports its synergy with sensitizing myeloma cells to anti CD38 monoclonal antibodies, proteasome inhibitors, and additivity with immunomodulatory treatments [16]. The unique selection of exportin 1 led to similar adverse events (anemia, thrombocytopenia, and neutropenia) to that of the Lonial et al. [15] study (mainly neutropenia). This can act as a future implication that inhibition of SLAMF7 and exportin 1 can potentiate hematological effects, but more importantly can potentially be used in combination with daratumumab (CD 38 monoclonal antibody) to maximize therapeutic benefit in myeloma patients.

Palumbo et al. [17] demonstrated a benefit in overall survival and progression-free survival using daratumumab with bortezomib, as well as dexamethasone for multiple myeloma. This stage three clinical trial was comprised of 498 multiple myeloma individuals currently being treated with daratumumab, that is a human IgG kappa monoclonal antibody which targets CD38, and that is highly conveyed in myeloma cells, inducing indirect and direct antimyeloma activity. Among the affected person population suffering from relapsed or relapsed and refractory multiple myeloma had been discovered to have substantially longer progression-free survival and reduced risk of disease progression (61.4% lower) with combination therapy comprising of daratumumab, bortezomib, and dexamethasone when compared to bortezomib and dexamethasone. The rates of a partial or good/complete response doubled those of the control group. The daratumumab group maintained longer periods of illness remission because of its longer median duration of time and response of subsequent antimyeloma treatment when compared with the control group. A phase two study of daratumumab in conjunction with dexamethasone and bortezomib versus bortezomib and dexamethasone resulted in a longer median progression-free survival of 9.7 months as compared to 6.9 months [17]. Higher incidences of infusion-related reactions and thrombocytopenia had been observed in the daratumumab group. Since the information evaluation is ongoing, no analyses based on baseline cytogenetic capabilities were achievable. The general survival benefit could not be evaluated in the daratumumab group due to the short follow-up period. Following the interim process, individuals in the control group had been permitted to get daratumumab, which would likely confound the last analysis of the overall survival. These results display an additive advantage of including daratumumab in conjunction with proteasome inhibitors/immunomodulatory agents as well as dexamethasone to treat multiple myeloma. Combination of CD 38 antibody with chemotherapy and proteasome inhibitor exhibited similar adverse effects to that of the SLAMF7 inhibition [15] and exportin 1 inhibition [16] that were thrombocytopenia and neutropenia. Future implications may want to test the effects of adding an exportin 1 inhibitor to CD 38 monoclonal antibody and chemotherapy in relapsed and refractory myeloma patients to enhance CD 38 inhibition.

Sequist et al. [21] mentioned that epidermal growth factor (EGFR) T790M mutation is present in 50 to 60% of resistant instances that features a median survival of less than 2 years after the mutation. A phase 1/2 clinical study comprising of 130 individuals had been treated with rociletinib in EGFR-mutated non-small-cell lung cancer. Rociletinib is a mutant selective covalent inhibitor of typical EGFR mutations, like, exon nineteen deletions, L858R, and T790M. There was sturdy tumor shrinkage in tumors conveying these activating mutations. The application of rociletinib in individuals with EGFR-mutated non-small-cell lung cancer resulted in sustained tumor responses. This study population had received a median number of four therapies just before rociletinib. Rociletinib selectively targets mutated EGFR and EGFR T790M mutations, resulting in a 59% response rate as well as prolonged disease management. Although greatest efficacy was observed in the EGFR T790M group, the group without the mutation displayed an anti-tumor response (29% response rate and median progression-free survival of 5.6 months). The proof of an EGFR T790M mutation could be affected by many variables, like, biological existence of T790M in some other tumors, precision of biopsy method in sampling the genuine tissue attributes, and genotype sensitivity. The main limitation of the analysis was the few individuals that have been treated with rociletinib. These outcomes of non-small-cell lung cancer with driver mutations have found great advantage with tyrosine kinase inhibition [21]. Interestingly, past therapeutics for non-small-cell lung cancer dealt with platinum-based chemotherapy, but treatment protocol has transitioned to identifying and treating driver mutations, which seems to be goal of any future implication. One would speculate that identifying mutations via diagnostic screening serves a great benefit to this subgroup.

The Reck et al. [18] scientific study shows that 23 to 28% of non-small-cell lung cancer patients have a programmed death ligand 1 (PD L1) expression on a minimum of 50% of tumor cells. This led to a stage three clinical trial comprising of 305 individuals that had been treated with pembrolizumab in PD L1( +) non-small-cell lung cancer. Pembrolizumab is a selective humanized monoclonal antibody against PD L1, preventing PD 1 engaging with PD-L1/PD-L2. This led to improved median progression-free as well as overall survival. About 1/3rd of individuals with non-small-cell lung cancer has stage three locally complex illness at diagnosis. The pembrolizumab versus chemotherapy for PD L1( +) non-small-cell lung cancer resulted in an overall advantage in response rate, longer period of reaction, lower frequency of therapy-related negative events, and longer general survival when administered pembrolizumab with a minimum of 50% PD L1 expression on tumor cells. This study stayed consistent with earlier trials consisting of individuals with untreated non-small-cell lung cancer and a PD L1 expression of a minimum of 50%. The previous trials are referred to this trial, KEYNOTE-010, KEYNOTE-001, KEYNOTE-042, and KEYNOTE 024 (trial related to this article) have resulted in pembrolizumab (humanized PD L1 antibody) simply being much more efficacious with respect to the abovementioned benefits as compared to regular chemotherapy. The adverse effects of pembrolizumab (humanized PD L1 antibody) were surprisingly different than the PD L1 therapy in treatment of triple-negative breast cancer, which shows similar combination therapy has varying adverse effects with respect to the malady. Future implications can deal with using various PD L1 antibodies that are used in other cancer treatment, such as, atezolizumab for triple-negative breast cancer, and observe if there is clinical benefit in non-small-cell lung cancer patients with/without PD L1 expression.

Antonia et al. [19] preclinical data recommended that radiotherapy and chemotherapy may enhance PD L1 expression in tumor cells in individuals with stage three non-small-cell lung cancer. This led to a stage three clinical trial comprising of 713 individuals receiving chemotherapy or combination durvalumab as well as chemotherapy for non-small-cell lung cancer. Durvalumab is a selective human IgG1 monoclonal antibody which blocks programmed death ligand 1 (PD L1) and CD80, resulting in T cells to wipe out tumor cells. Durvalumab combination with chemotherapy has demonstrated a considerable advantage in progression-free survival (11 months longer) in non-small-cell lung cancer when compared to chemotherapy. The vast majority of the affected person population had a PD L1 expression of 25% or even less frequently and that the distinction in progression-free survival was found across all subgroups. Secondary endpoint, objective response rate was 12.4% higher in the durvalumab group than placebo. The durvalumab group had 72.8% of patient response at 12 and 18 months as compared to 56.1% as well as 46.8% in the placebo. A favorable impact on metastase frequency was observed in the durvalumab group. This study, similar to the Reck et al. [18] trial-targeted PD L1, but had different adverse reactions compared to each other, as well as the PD L1 antibody therapy in triple-negative breast cancer. The concept of using chemotherapy/radiotherapy can be similar to an extent to that of exportin 1 enhancing CD 38 expression in multiple myeloma [16], but instead PD L1 expression is amplified that can allow for a more effective response rate when using a PD L1 antibody-like durvalumab in non-small-cell lung cancer. Future implications can lead to observing which tumor markers are amplified with chemotherapy use and then can be selectively targeted with immunotherapy for a more significant response.

## Conclusion

In essence, one can determine that using immunotherapy in combination with chemotherapy is far superior to that of chemotherapy in respect to overall/progression-free survival, response rate, duration of response, clinical benefit, and invasive-disease-free survival in triple-negative/HER2 2( +) breast cancer, multiple myeloma, and non-small-cell lung cancer.

## Supplementary information

**Additional file 1.**

## Data Availability

The datasets generated and/or analyzed during the current study are not publicly available due to HIPAA but are available from the corresponding author on reasonable request, if permissible by patient.
