# Supplementary material for: Combination of chemotherapeutic agents and biological response modifiers (immunotherapy) in triple-negative/Her2( +) breast cancer, multiple myeloma, and non-small-cell lung cancer
Source: J Egypt Natl Canc Inst. 2023 Jan 2;34:58. doi: 10.1186/s43046-022-00159-8 (PMC13314267; doi:10.1186/s43046-022-00159-8)
Supplement: Supplementary file 1 — Additional file 1. [file 43046_2022_159_MOESM1_ESM.docx]

**Appendix**

**Figure 1A.** Patient selection criteria (Schmid et al., 2018). https://www.nejm.org/doi/full/10.1056/nejmoa1809615


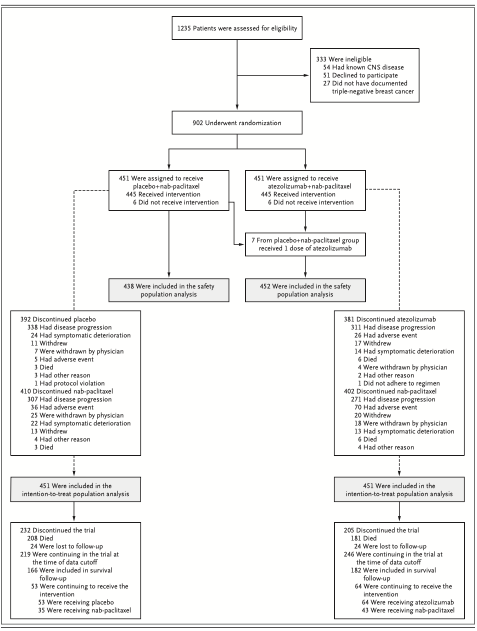


**Table 1A.** Characteristics of patient baseline (Schmid et al., 2018). https://www.nejm.org/doi/full/10.1056/nejmoa1809615


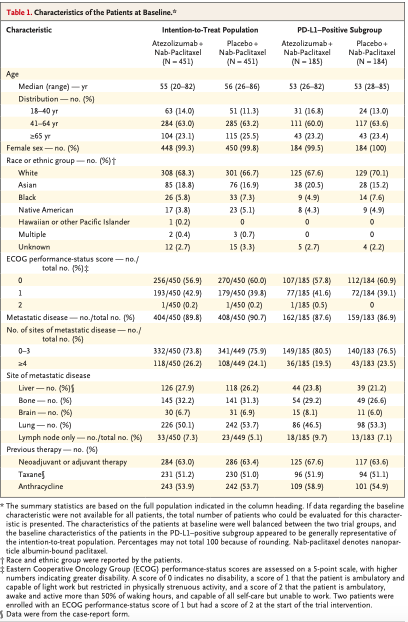


**Table 2A.** Secondary efficacy endpoints (Schmid et al., 2018). https://www.nejm.org/doi/full/10.1056/nejmoa1809615

***
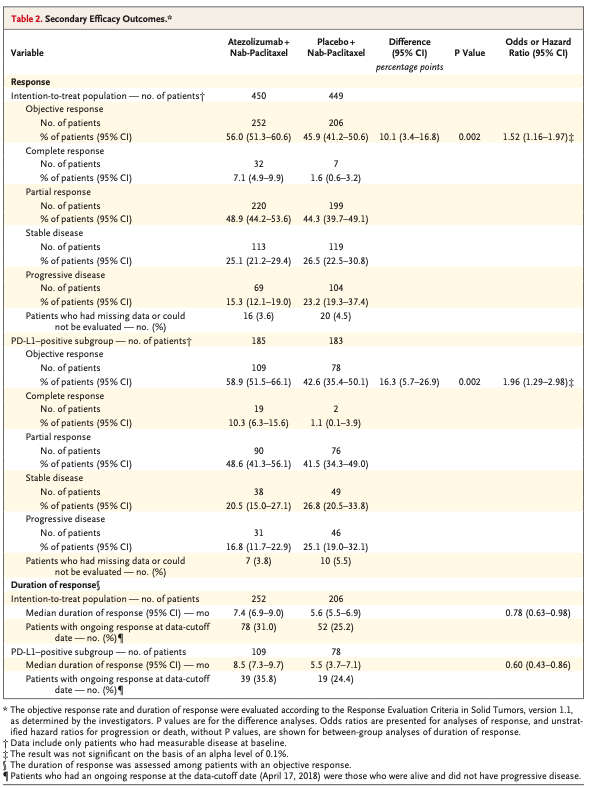
***

**Figure 2A.** Kaplan-Meier plot of progression-free/overall survival in PD-L1 positive groups (Schmid et al., 2018). https://www.nejm.org/doi/full/10.1056/nejmoa1809615

***
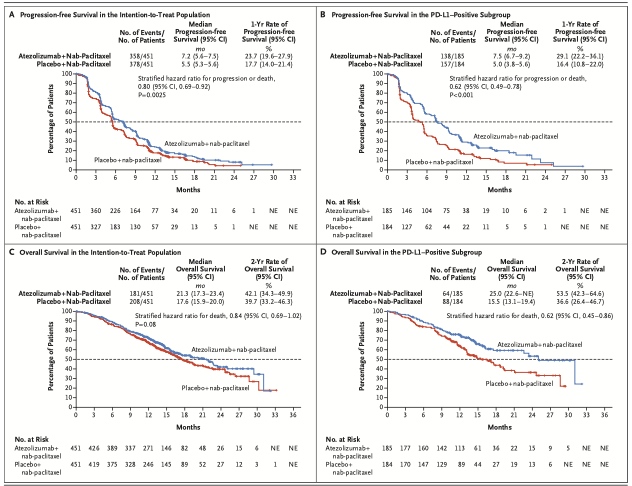
***

**Figure 1B.** Graphs with respect to tumor size, objective response rate and progression-free survival (Bardia et al., 2019). https://www.nejm.org/doi/pdf/10.1056/NEJMoa1814213

***
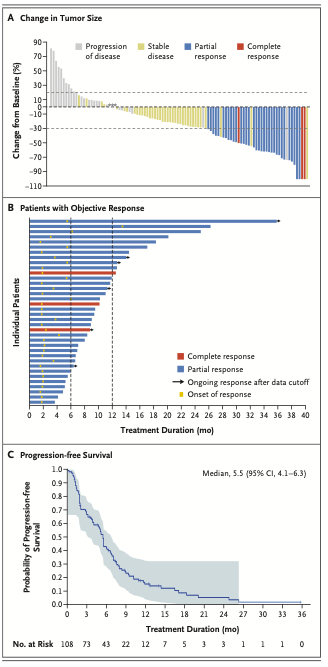
***

**Figure 2B.** Duration of treatment in Sacituzumab group versus patients with previous anticancer treatment (Bardia et al., 2019). https://www.nejm.org/doi/pdf/10.1056/NEJMoa1814213

***
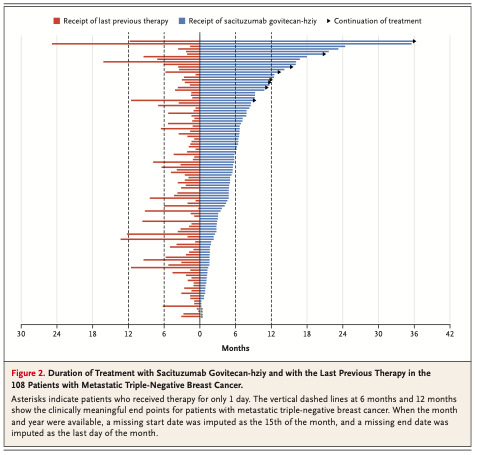
***

**Figure 3B.** CT scan of before and after Durvalumab treatment (Al sayed., et al 2019). https://www.amjcaserep.com/download/index/idArt/918770

***
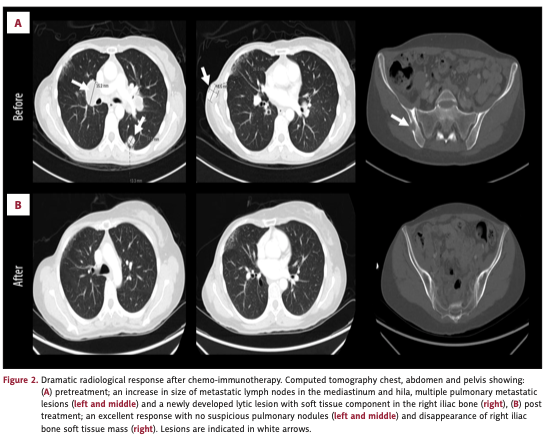
***

**Figure 4B.** Before and after chemo-immunotherapy bone scan (Al sayed., et al 2019). https://www.amjcaserep.com/download/index/idArt/918770

***
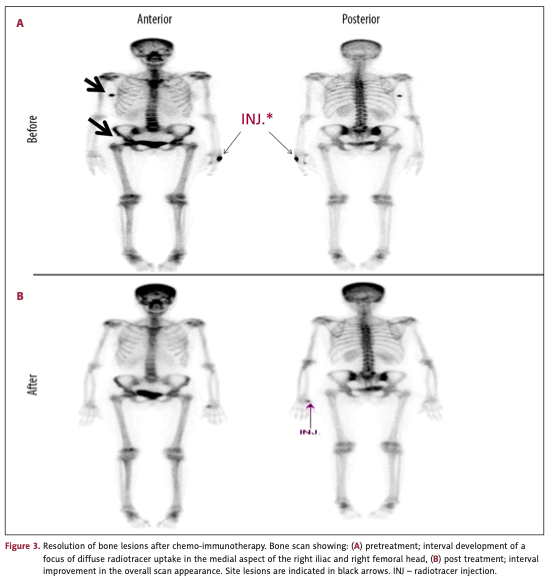
***

**Figure 1C.** Kaplan-Meier plot of invasive-disease-free survival (Von Minckwitz., et al 2018). https://www.nejm.org/doi/full/10.1056/NEJMoa1703643

***
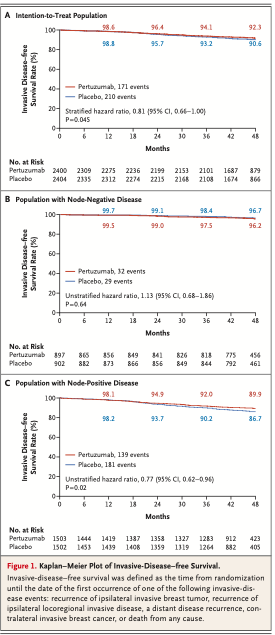
***

**Figure 1D.** Kaplan-Meier plot of progression free survival of Elotuzumab group versus control (Lonial et al., 2015). https://www.nejm.org/doi/full/10.1056/NEJMoa1505654


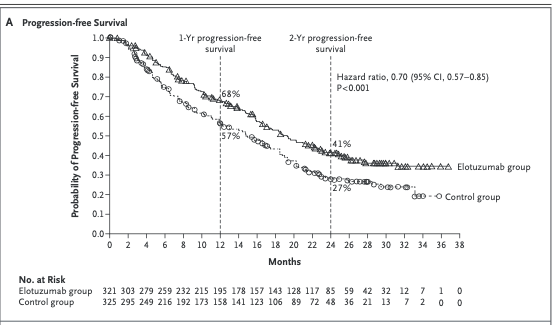


**Figure 1E.** Kaplan-Meier plot of progression-free/overall response and survival (Chari et al., 2019). https://www.nejm.org/doi/full/10.1056/NEJMoa1903455

***
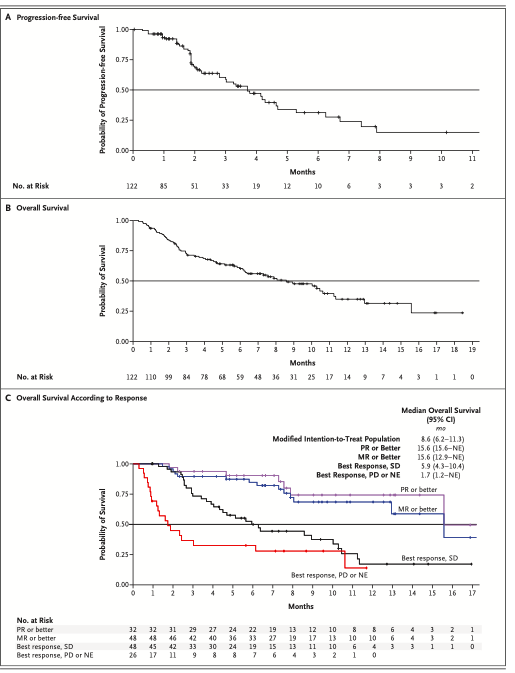
***

**Figure 1F.** Kaplan-Meier plot of progression-free survival and time to disease progression (Palumbo et al., 2016). https://www.nejm.org/doi/full/10.1056/nejmoa1606038

***
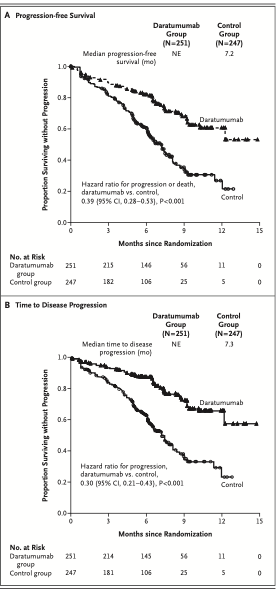
***

**Figure 1G.** Bar graph denoting change in tumor burden based on T790M expression (Sequist., et al 2015). https://www.nejm.org/doi/full/10.1056/NEJMoa1413654

**
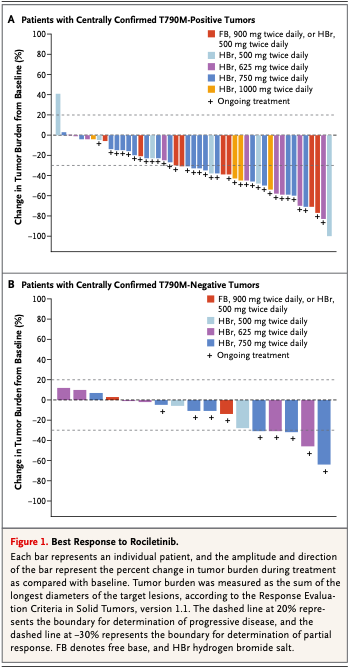
**

**Figure 1H.** Kaplan-Meier plot of Pembrolizumab versus chemotherapy group regarding progression-free survival (Reck., et al 2016). https://www.nejm.org/doi/full/10.1056/nejmoa1606774

***
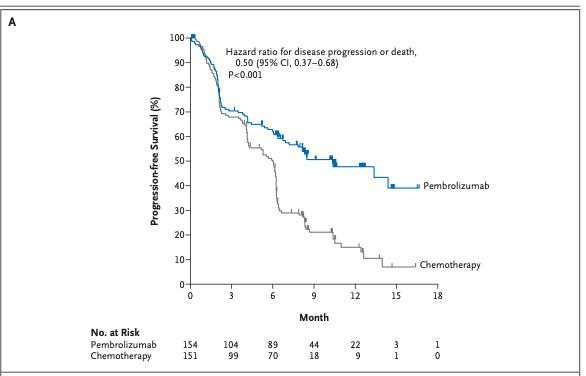
***

**Figure 2H.** Kaplan-Meier plot with overall survival in Pembrolizumab versus chemotherapy group regarding overall survival (Reck., et al 2016). https://www.nejm.org/doi/full/10.1056/nejmoa1606774

***
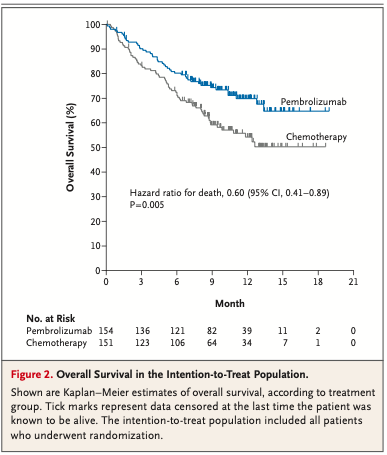
***

**Figure 1I.** Kaplan-Meier plot of progression-free survival in Durvalumab versus placebo group (Antonia., et al 2017). https://www.nejm.org/doi/pdf/10.1056/NEJMoa1709937

**
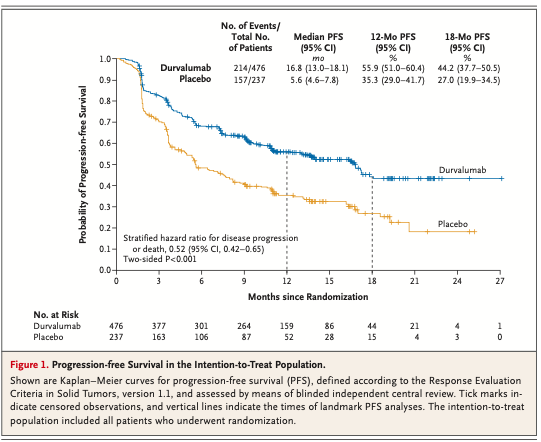
**

**Evidence table**

******

***DECLARATIONS***

Ethics approval and consent to participate: Not applicable

Consent for publication: Not applicable

Availability of data and material: The datasets generated and/or analyzed during the current study are publicly available through google-scholar and corresponding author, upon request.

Competing interests: None

Funding: Not applicable

Authors' contributions: ‘WM’ was a major contributor in the writing of the manuscript. ‘HN’ oversaw the patient with the attending physician and contributed to the editing of the paper. ‘A.A.C’ helped with the interpretation of the patient data. All authors approved the final manuscript.

Acknowledgements: Not applicable

***References***

Al Sayed, A. D., Elshenawy, M. A., Tulbah, A., Al-Tweigeri, T., & Ghebeh, H. (2019). Complete Response of Chemo-Refractory Metastatic Metaplastic Breast Cancer to Paclitaxel-Immunotherapy Combination. *The American journal of case reports*, *20*, 1630–1635. https://doi.org/10.12659/AJCR.918770

Antonia, S. J., Villegas, A., Daniel, D., Vicente, D., Murakami, S., Hui, R., ... & Cho, B. C. (2017). Durvalumab after chemoradiotherapy in stage III non–small-cell lung cancer. *New England Journal of Medicine*, *377*(20), 1919-1929.

Bardia, A., Mayer, I. A., Vahdat, L. T., Tolaney, S. M., Isakoff, S. J., Diamond, J. R., ... & Shah, N. C. (2019). Sacituzumab govitecan-hziy in refractory metastatic triple-negative breast cancer. *New England Journal of Medicine*, *380*(8), 741-751.

Borghaei, H., Paz-Ares, L., Horn, L., Spigel, D. R., Steins, M., Ready, N. E., ... & Brahmer, J. R. (2015). Nivolumab versus docetaxel in advanced nonsquamous non–small-cell lung cancer. *New England Journal of Medicine*, *373*(17), 1627-1639.

Chari, A., Vogl, D. T., Gavriatopoulou, M., Nooka, A. K., Yee, A. J., Huff, C. A., ... & Dimopoulos, M. (2019). Oral Selinexor–Dexamethasone for Triple-Class Refractory Multiple Myeloma. *New England Journal of Medicine*, *381*(8), 727-738.

Dimopoulos, M. A., Dytfeld, D., Grosicki, S., Moreau, P., Takezako, N., Hori, M., ... & San-Miguel, J. (2018). Elotuzumab plus pomalidomide and dexamethasone for multiple myeloma. *New England Journal of Medicine*, *379*(19), 1811-1822.

Hellmann, M. D., Ciuleanu, T. E., Pluzanski, A., Lee, J. S., Otterson, G. A., Audigier-Valette, C., ... & Paz-Ares, L. (2018). Nivolumab plus ipilimumab in lung cancer with a high tumor mutational burden. *New England Journal of Medicine*, *378*(22), 2093-2104.

Key Statistics for Multiple Myeloma. (2020). Retrieved December 04, 2020, from https://www.cancer.org/cancer/multiple-myeloma/about/key-statistics.html

Lonial, S., Dimopoulos, M., Palumbo, A., White, D., Grosicki, S., Spicka, I., ... & Belch, A. (2015). Elotuzumab therapy for relapsed or refractory multiple myeloma. *New England Journal of Medicine*, *373*(7), 621-631.

*Lung Cancer Survival Rates | 5-Year Survival Rates for Lung Cancer*. (2020). American Cancer Society. https://www.cancer.org/cancer/lung-cancer/detection-diagnosis-staging/survival-rates.html

Mateos, M. V., Dimopoulos, M. A., Cavo, M., Suzuki, K., Jakubowiak, A., Knop, S., ... & San-Miguel, J. (2018). Daratumumab plus bortezomib, melphalan, and prednisone for untreated myeloma. *New England Journal of Medicine*, *378*(6), 518-528.

O'Shaughnessy, J., Osborne, C., Pippen, J. E., Yoffe, M., Patt, D., Rocha, C., ... & Bradley, C. (2011). Iniparib plus chemotherapy in metastatic triple-negative breast cancer. *New England Journal of Medicine*, *364*(3), 205-214.

Palumbo, A., Chanan-Khan, A., Weisel, K., Nooka, A. K., Masszi, T., Beksac, M., ... & Mark, T. M. (2016). Daratumumab, bortezomib, and dexamethasone for multiple myeloma. *New England Journal of Medicine*, *375*(8), 754-766.

Park, J. W., Liu, M. C., Yee, D., Yau, C., van’t Veer, L. J., Symmans, W. F., ... & Berry, D. A. (2016). Adaptive randomization of neratinib in early breast cancer. *New England Journal of Medicine*, *375*(1), 11-22.

Raje, N., Berdeja, J., Lin, Y., Siegel, D., Jagannath, S., Madduri, D., ... & Kochenderfer, J. N. (2019). Anti-BCMA CAR T-cell therapy bb2121 in relapsed or refractory multiple myeloma. *New England Journal of Medicine*, *380*(18), 1726-1737.

Reck, M., Rodríguez-Abreu, D., Robinson, A. G., Hui, R., Csőszi, T., Fülöp, A., ... & O’Brien, M. (2016). Pembrolizumab versus chemotherapy for PD-L1–positive non–small-cell lung cancer. *New England Journal of Medicine*, *375*(19), 1823-1833.

Schmid, P., Adams, S., Rugo, H. S., Schneeweiss, A., Barrios, C. H., Iwata, H., ... & Henschel, V. (2018). Atezolizumab and nab-paclitaxel in advanced triple-negative breast cancer. *New England Journal of Medicine*, *379*(22), 2108-2121.

Sequist, L. V., Soria, J. C., Goldman, J. W., Wakelee, H. A., Gadgeel, S. M., Varga, A., ... & Aisner, D. L. (2015). Rociletinib in EGFR-mutated non–small-cell lung cancer. *New England Journal of Medicine*, *372*(18), 1700-1709.

*Triple-negative Breast Cancer | Details, Diagnosis, and Signs*. (2020). American Cancer Society. https://www.cancer.org/cancer/breast-cancer/understanding-a-breast-cancer-diagnosis/types-of-breast-cancer/triple-negative.html

Von Minckwitz, G., Eidtmann, H., Rezai, M., Fasching, P. A., Tesch, H., Eggemann, H., ... & Untch, M. (2012). Neoadjuvant chemotherapy and bevacizumab for HER2-negative breast cancer. *New England Journal of Medicine*, *366*(4), 299-309.

Von Minckwitz, G., Procter, M., De Azambuja, E., Zardavas, D., Benyunes, M., Viale, G., ... & Knott, A. (2017). Adjuvant pertuzumab and trastuzumab in early HER2-positive breast cancer. *New England Journal of Medicine*, *377*(2), 122-131.
